# Compliance of the cerebrospinal space: comparison of three methods

**DOI:** 10.1007/s00701-021-04834-y

**Published:** 2021-04-14

**Authors:** Agnieszka Kazimierska, Magdalena Kasprowicz, Marek Czosnyka, Michał M. Placek, Olivier Baledent, Peter Smielewski, Zofia Czosnyka

**Affiliations:** 1 Department of Biomedical Engineering, Faculty of Fundamental Problems of Technology, Wroclaw University of Science and Technology, Wroclaw, Poland; 2 Brain Physics Laboratory, Division of Neurosurgery, Department of Clinical Neurosciences, Addenbrooke’s Hospital, University of Cambridge, Cambridge, UK; 3 Institute of Electronic Systems, Faculty of Electronics and Information Technology, Warsaw University of Technology, Warsaw, Poland; 4 Department of Medical Image Processing, CHU Amiens, University of Picardy Jules Verne, Amiens, France

**Keywords:** Intracranial pressure, Cerebrospinal compliance, Infusion test, Cerebral arterial blood volume, Pulse waveform

## Abstract

**Background:**

Cerebrospinal compliance describes the ability of the cerebrospinal space to buffer changes in volume. Diminished compliance is associated with increased risk of potentially threatening increases in intracranial pressure (ICP) when changes in cerebrospinal volume occur. However, despite various methods of estimation proposed so far, compliance is seldom used in clinical practice. This study aimed to compare three measures of cerebrospinal compliance.

**Methods:**

ICP recordings from 36 normal-pressure hydrocephalus patients who underwent infusion tests with parallel recording of transcranial Doppler blood flow velocity were retrospectively analysed. Three methods were used to calculate compliance estimates during changes in the mean ICP induced by infusion of fluid into the cerebrospinal fluid space: (a) based on Marmarou’s model of cerebrospinal fluid dynamics (C_CSF_), (b) based on the evaluation of changes in cerebral arterial blood volume (C_CaBV_), and (c) based on the amplitudes of peaks P1 and P2 of ICP pulse waveform (C_P1/P2_).

**Results:**

Increase in ICP caused a significant decrease in all compliance estimates (*p* < 0.0001). Time courses of compliance estimators were strongly positively correlated with each other (group-averaged Spearman correlation coefficients: 0.94 [0.88–0.97] for C_CSF_ vs. C_CaBV_, 0.77 [0.63–0.91] for C_CSF_ vs. C_P1/P2_, and 0.68 [0.48–0.91] for C_CaBV_ vs. C_P1/P2_).

**Conclusions:**

Indirect methods, C_CaBV_ and C_P1/P2_, allow for the assessment of relative changes in cerebrospinal compliance and produce results exhibiting good correlation with the direct method of volumetric manipulation. This opens the possibility of monitoring relative changes in compliance continuously.

## Introduction

In adults, the skull is a closed non-distensible box filled with three volume components: brain tissue, cerebral blood, and cerebrospinal fluid (CSF) [16]. According to the Monro–Kellie doctrine, in the long-term—and under normal conditions—increases in the volume of one component are compensated by decreases in the volume of another, maintaining the total volume at an approximately constant level [20]. However, in the short-term scale, considering pulsatile blood flow, for a part of the cardiac cycle, total blood volume increases and then decreases, leaving total volume changes equal to zero [2]. This produces rhythmic instability of the intracranial pressure (ICP) signal, known as ‘pulse waveform’. The ability of the cerebrospinal space to accommodate changes in volume is quantified by a parameter called compliance which links changes in volume with corresponding changes in pressure [23]. Low compliance puts the system at risk of disproportionately large increases in ICP for even small increases in intracranial volume, potentially leading to intracranial hypertension [21]. To measure cerebrospinal compliance, external gradual volume addition into the cerebrospinal space is needed, which is a limitation preventing continuous monitoring. We aimed to examine and compare three methods of assessment of cerebrospinal compliance. One is a ‘golden standard’ based on external volume load, and two others rely on the evaluation of changes related to blood stroke volume over heart period. These two, if they are linked to ‘gold standard’ compliance, would allow for continuous monitoring in various clinical scenarios, for instance, hydrocephalus ICP monitoring or traumatic head injury (TBI) and subarachnoid haemorrhage neuro-intensive care monitoring.

The infusion study is a type of volume–pressure test based on the injection of additional volume into the cerebrospinal space that allows for the estimation of compensatory parameters, including cerebrospinal compliance, from a mathematical model of CSF circulation [13]. In the present work, we compared compliance estimates obtained with the model of CSF dynamics (the ‘gold standard’ method) during changes in the mean ICP induced by constant rate infusion of fluid into the CSF space in normal-pressure hydrocephalus (NPH) patients with two other measures: based on the estimation of changes in cerebral blood volume (CBV) in each cardiac cycle and based on the analysis of changes in the ratio of characteristic peaks P1 and P2 of ICP pulse waveform. The study offers new insight into the feasibility of using the P1/P2 ratio and evaluation of changes in CBV as a tool for continuous monitoring of compliance of the cerebrospinal space.

## Materials and methods

### Data acquisition

Data from patients who underwent infusion studies with simultaneous recording of ICP and cerebral blood flow velocity (CBFV) signals at Addenbrooke’s Hospital (Cambridge, UK) between 1993 and 1998 were selected for retrospective analysis in this study. Selection of patients was made on a basis of good quality of ICP pulse waveform and CBFV recording, and only those tests where the shape of the pulse waveform of ICP presented both P1 and P2 peaks were classified as suitable for analysis. Out of the full group of 72 considered recordings, 36 were chosen. Sixteen recordings (22%) were excluded because of rounded ICP pulse waveform that did not allow for the identification of both characteristic peaks, and 20 recordings (28%) were excluded due to insufficient quality of either ICP or CBFV signals. The data were collected as part of routine clinical investigation following diagnosis of NPH. Ethics committee approval to record ICP and CBFV using transcranial Doppler (TCD) ultrasonography was obtained.

The computerized infusion test was performed with two hypodermic needles (25 gauges). One of the needles was used for ICP measurement and connected to a pressure transducer through a saline-filled tube. The second needle was used for infusion and connected to an infusion pump containing a pressure amplifier (Simonsen & Will, Sidcup, UK). The infusion of normal saline into the CSF space was performed at the rate of 1.5 ml/min. The test began with 10 min of baseline recording of ICP before the start of the infusion. Infusion continued until the increase in ICP reached either the plateau phase or the maximum acceptable level of 40 mm Hg, and the recordings were further collected until ICP returned to baseline level. CBFV in the middle cerebral artery was monitored during the test using a TCD unit (Neuroguard; MedaSonics, Fremont, CA, USA) with a 2-MHz probe locked in a stable position using a commercially available fixation system. Twenty-six recordings included also arterial blood pressure (ABP) monitored noninvasively using a photoplethysmographic system (Finapres; Finapres Medical Systems, the Netherlands).

**Fig. 1:**
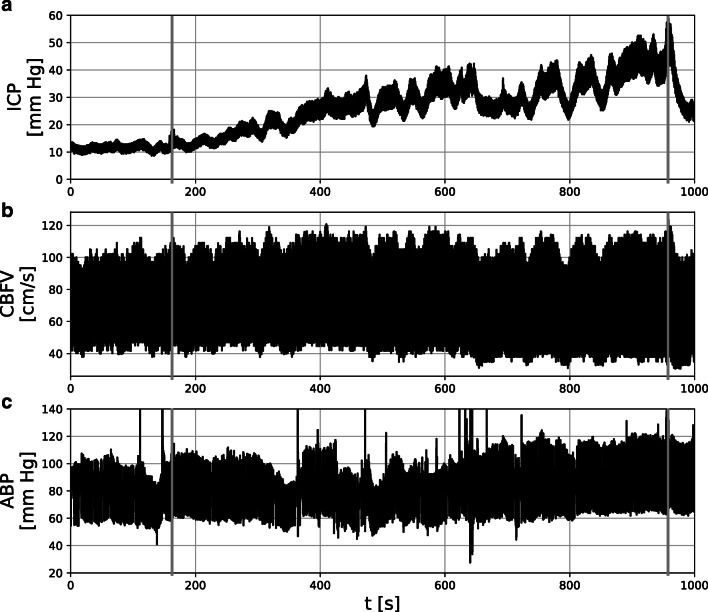
Illustrative example of signals recorded during the infusion test for a single patient. Grey vertical lines indicate the start and end of constant rate (1.5 ml/min) infusion. **a** Intracranial pressure (ICP). **b** Cerebral blood flow velocity (CBFV). **c** Arterial blood pressure (ABP)

An analogue-to-digital converter (DT 2814; Data Translation, Marlboro, USA) connected to an IBM AT laptop computer (Amstrad ALT 386 SX; Amstrad, Brentwood, UK) was used for the collection of data from the pressure monitors and the TCD system. The signals were sampled at frequency ranging from 30 to 50 Hz using custom software for waveform recording (WREC; W. Zabolotny, Warsaw University of Technology, Warsaw, Poland). An illustrative example of recorded signals is presented in Fig. 1.

### Three methods of estimation of cerebrospinal compliance

The relationship between pressure and volume in the intracranial space is described by an exponential function known as the pressure–volume curve, which shows that changes in ICP increase progressively with the mean ICP [29]. Traditionally, measurement of the pressure response requires external manipulation of intracranial volume, such as bolus injection or constant rate infusion [18, 24]. A different approach to compliance estimation relies on the possibility of measuring changes in CBV alongside ICP. However, a method based on magnetic resonance imaging [1], although potentially accurate, is currently only applicable to the evaluation of instantaneous, or ‘snapshot’, values, and continuous estimation of changes in CBV using TCD recordings of CBFV in large cerebral arteries [19] does not allow for calibration of obtained values due to the unknown cross-sectional area of insonated vessels.

**Fig. 2:**
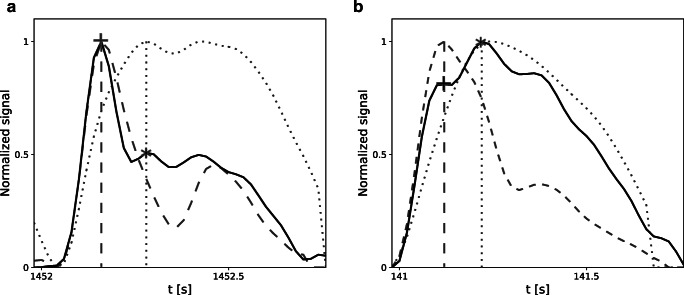
Illustrative examples of intracranial pressure (ICP) pulse waveforms from two different patients. Location of peaks P1 and P2 is indicated by cross (P1) and star (P2) signs. ICP signals are plotted with solid black lines. Additional signals used in the process of peak detection are plotted as dashed (arterial blood pressure (ABP)) and dotted (cerebral arterial blood volume (CaBV)) lines. All signals are normalized and aligned with regard to pulse onset location. Vertical lines indicate the correlation between the position of peaks P1 and P2 and the local maxima of the ABP (dashed line) and CaBV (dotted line) waveforms, respectively

It has also been suggested that information about cerebral compliance may be extracted from the pulse waveform of ICP itself [6]. Three major features commonly used to characterize ICP pulse waveform are peaks P1, P2, and P3 [16]. Although the precise origin of those peaks is not universally agreed upon, the shape of the ICP waveform is believed to arise from changes in both ABP and CBV [7]. Under normal circumstances, P1 dominates over the other two peaks, resulting in a saw-tooth appearance of the waveform (Fig. 2a). As the mean ICP increases, so does the amplitude of characteristic peaks. However, the change is not uniform, and rising prominence of P2 (Fig. 2b) eventually leads to a rounded or triangular waveform with indistinguishable P1 and P3 [9, 10]. Given the relatively larger changes in the magnitude of the P2 component, it has been suggested that the ratio of peak amplitudes, P1/P2, may provide information about cerebral compliance. Still, despite its potential as a means for long-term monitoring of the state of the cerebrospinal space, very little attention has been devoted to the application of this parameter in the evaluation of cerebral compensatory reserve.

In the present study, three methods were used to obtain estimates of cerebrospinal compliance: (a) based on a model of CSF dynamics, (b) based on evaluation of changes in CBV, and (c) based on the ICP pulse waveform. Assessment of model-based parameters describing CSF dynamics (a) was performed using built-in algorithms of specialized software (ICM+; Cambridge Enterprise, Cambridge, UK). All other analyses were performed using programmes custom-written in Python 3.8.

#### Model of cerebrospinal fluid dynamics

The first estimate of cerebrospinal compliance (denoted C_CSF_ from ‘cerebrospinal fluid dynamics’) was obtained from a mathematical model of CSF volume–pressure compensation [12]. Under normal conditions, in the absence of long-term fluctuations in cerebral blood volume, it is assumed that the production of CSF is balanced by its storage and reabsorption. Including external CSF infusion, this can be described by the following relationship:

1 $$ \mathrm{production}\ \mathrm{of}\ \mathrm{CSF}+\mathrm{infusion}=\mathrm{storage}\ \mathrm{of}\ \mathrm{CSF}+\mathrm{reabsorption}\ \mathrm{of}\ \mathrm{CSF} $$

The rate of CSF production is assumed to be constant. *I*(*t*) is the rate of the external volume infusion (e.g.,1.5 ml/min in the present study). The rate of CSF storage (*I*_s_) is proportional to cerebrospinal compliance (*C*) and depends on the derivative of CSF pressure (*P*) over time (*dP/dt*):

2 $$ {I}_s=C\frac{dP}{dt} $$

Compliance is in turn inversely proportional to the gradient of CSF pressure and reference pressure (*P*_0_):

3 $$ C=\frac{1}{E\left(P-{P}_o\right)} $$

where *E* is cerebral elasticity. The rate of CSF reabsorption (*I*_r_) is proportional to the gradient between CSF pressure *P* and pressure in the sagittal sinuses (*P*_ss_):

4 $$ {I}_r=\frac{P-{P}_{ss}}{R_{out}} $$

where *R*_out_ describes the resistance to CSF outflow. Relationship (3) is considered valid only above certain pressure level described as ‘lower breakpoint pressure’. It is the pressure above which the cerebrospinal pressure–volume curve becomes exponential. Below this pressure, the pressure–volume relationship is linear. The combination of Eqs. (1) to (4) produces the final equation:

5 $$ \frac{1}{E\left(P-{P}_0\right)}\frac{dP}{dt}+\frac{P-{P}_b}{R_{out}}=I(t) $$

where *P*_b_ is the baseline pressure.

Parameters *R*_out_, *E*, and *P*_0_ were estimated for ICP recordings used in this study based on the analytical solution of Eq. (5) for constant rate infusion. *E* and *P*_0_ were then used to obtain estimates of cerebrospinal compliance over the course of the infusion test from Eq. (3). To maintain cohesion with the other two pulse-by-pulse methods of estimation, the mean ICP over each cardiac cycle was taken as *P*(*t*).

#### Evaluation of pulsatile changes in cerebral blood volume

The second estimate of cerebrospinal compliance (denoted C_CaBV_ from ‘cerebral arterial blood volume’) was obtained from a mathematical model of cerebral blood circulation using the approach described by Kim et al. [19]. The change in CBV over a single cardiac cycle can be expressed as the difference between arterial inflow (CBF_a_) and venous outflow (CBF_v_):

6 $$ \Delta  CBV(t)={\int}_{t_0}^t\left({CBF}_a(s)-{CBF}_v(s)\right) ds $$

where *t*_0_, *t* are the beginning and end of the cardiac cycle, respectively, and *s* is the variable of integration. Given the assumption that venous outflow has low pulsatility compared to arterial inflow, the former can be approximated by a constant flow equal to averaged arterial inflow:

7 $$ \Delta  {C}_a BV(t)={\int}_{t_0}^t\left({CBF}_a(s)-{meanCBF}_a\right)d\mathrm{s} $$

Furthermore, cerebral arterial blood flow can be expressed as cerebral blood flow velocity multiplied by cross-sectional area of the vessel. Using TCD recordings of CBFV in cerebral arteries and assuming that the cross-sectional area of insonated vessel remains constant, Eq. (7) can be approximated as

8 $$ \Delta  {C}_a BV(n)={S}_a\cdotp {\sum}_{i=1}^n\left({CBFV}_a(i)- mean{CBFV}_a\right)\Delta  t(i) $$

where *n* is the number of samples from the beginning of the cardiac cycle, *Δt* is the time interval between two consecutive samples, and *S*_a_ is the unknown cross-sectional area of insonated vessel.

Based on pulsatile signals CaBV and ICP, cerebrospinal compliance (*C*) can be estimated as

9 $$ C=\frac{AMP_{C_a BV}}{AMP_{ICP}} $$

where *AMP*_CaBV_ and *AMP*_ICP_ are the amplitudes of fundamental components of CaBV and ICP, respectively. However, due to unknown *S*_a_, resulting values of *C* cannot be calibrated in units of ml per mm Hg.

In this study, CaBV was estimated from Eq. (8) based on CBFV recordings in the middle cerebral artery. Peak-to-peak amplitudes of CaBV and ICP in each cardiac cycle were then used to obtain pulse-by-pulse estimates of cerebrospinal compliance using Eq. (9).

#### ICP waveform analysis

The third estimate of cerebrospinal compliance (denoted C_P1/P2_ from the P1/P2 amplitude ratio) was based on the analysis of the ICP pulse waveform. Prior to the analysis, ICP, CBFV, and ABP (where available) signals were filtered using a low-pass filter with the cut-off frequency of 10 Hz. Individual pulses in the ICP signal were identified using a modified Scholkmann algorithm [4]; corresponding sections of CBFV and ABP signals were extracted based on pulse onset locations from ICP. For the purpose of peak annotation, each pulse was normalized and linearly detrended, and the three signals were aligned with regard to pulse onset in order to remove the phase shift resulting from distance between measurement sites.

A semi-automated algorithm based on the detection of the local maxima was used for peak identification. The algorithm incorporated information about the local maxima of ABP and CaBV (derived from the CBFV signal using Eq. (8)), taking into account observations from previous studies which showed that P1 is associated with the propagation of the pulse pressure wave through cerebral arteries and usually coincides with the systole of ABP, while P2 is derived from arterial blood volume load and coincides with the maximum of CaBV [8, 15]. In each pulse waveform, the local maxima of ICP corresponding to the position of maxima in ABP and CaBV were selected as candidates for P1 and P2, respectively. In the absence of the ABP signal, the first maximum of the CBFV signal was used instead to identify P1 candidates. Full detection results were reviewed and manually corrected in cases of insufficient detection accuracy, particularly in pathologically rounded waveforms. Pulses with distorted ICP waveform or unidentifiable P1 and P2 were excluded from further analyses. Illustrative examples of ICP pulse waveforms with peak annotations are presented in Fig. 2.

The amplitude of peaks P1 and P2 in each pulse was calculated as the vertical distance to the preceding local minimum identified as pulse onset. Pulse-by-pulse P1/P2 amplitude ratio was then used as a compliance estimate.

### Statistical analysis

Statistical analyses were performed using Python 3.8 with the built-in methods included in the SciPy 1.5.0 package. The Shapiro–Wilk test with a significance level of 0.05 was used to assess normality of data distributions. Upon rejection of the normality hypothesis for most of the analysed variables, non-parametric methods were chosen to analyse the relationship between compliance estimates. Time courses of compliance estimates obtained for individual patients with each of the three methods were compared with each other and with ICP using the Spearman correlation coefficient. To reduce the effect of difference in time scales used in compliance estimation, 30-pulse moving averages were used.

In order to compare the ‘high’ and ‘low’ compliance states reflecting the baseline and plateau phase of the infusion test, 1-minute-long fragments of the recordings where all three compliance estimates could be obtained were selected manually. Due to significant distortion of the ICP pulse waveform during baseline in a number of recordings, the initial stage of infusion was selected instead as baseline. Two patients for whom baseline values were not available either due to low quality of the ICP signal or the lower breakpoint pressure limit used in estimation of C_CSF_ were excluded from this part of the analysis. Values averaged over the baseline and plateau phases of the infusion test were compared using the Wilcoxon signed rank test. The same methods were used to determine the significance of changes in amplitude of peaks P1 and P2.

A significance level of 0.05 was assumed in all statistical tests. All group-averaged values are presented as median [first quartile–third quartile].

## Results

### Patient characteristics

The mean age of the patients was 54 years (range, 27–76 years). The patients showed ventricular dilation marked by increased bicaudate index (mean, 0.27; range, 0.14–0.39), and 14% showed white matter ischemia. Initial ICP in the group was 8.7 [3.8–11.4] mm Hg. Group-averaged R_out_ and elasticity were 12.1 [8.9–15.8] mm Hg/(ml/min) and 0.19 [0.14–0.33] ml^−1^, respectively.

### Amplitude of peaks P1 and P2 during changes in mean ICP

**Fig. 3:**
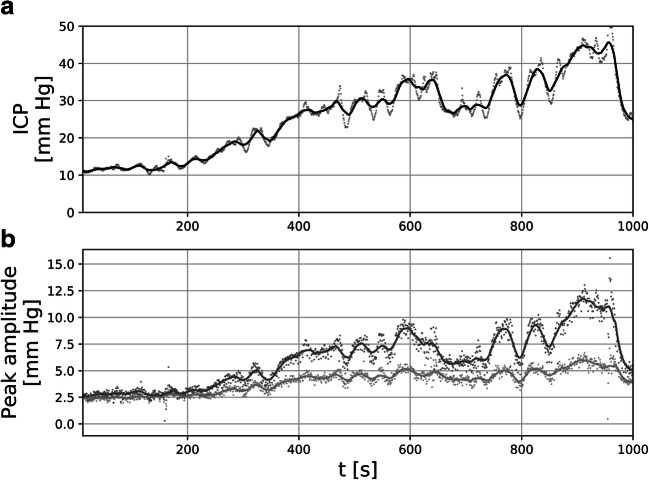
Illustrative example of time courses of amplitude of peaks P1 and P2 for a single patient. Full pulse-by-pulse time courses are presented as dots while 30-pulse moving averages are presented as solid lines. **a** Mean intracranial pressure (ICP). **b** Amplitude of peaks P1 (light grey symbols) and P2 (dark grey symbols) of the ICP pulse waveform

**Fig. 4:**
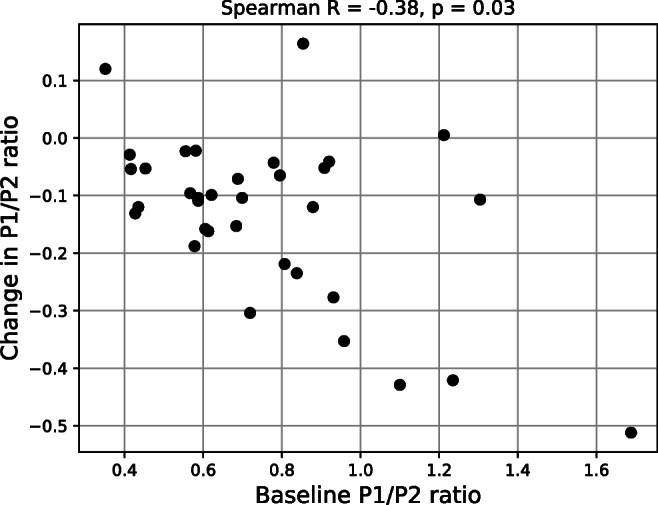
Relationship between baseline P1/P2 ratio and change in P1/P2 ratio between baseline and plateau phases of infusion test. Values above the scatter plot indicate Spearman correlation coefficient and its *p*-value

**Fig. 5:**
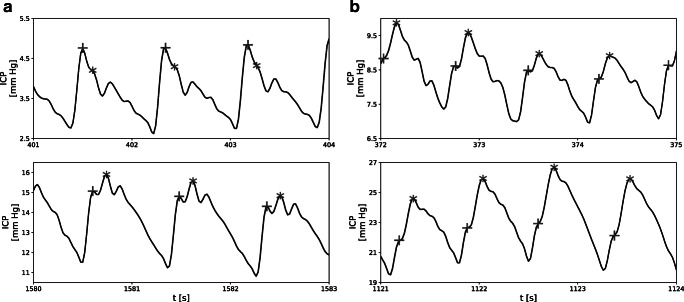
Illustrative examples of changes in intracranial pressure (ICP) pulse waveform between baseline and plateau phases of infusion test. Baseline and plateau phases are presented in the top and bottom plots, respectively. Location of peaks P1 and P2 is indicated by cross (P1) and star (P2) signs. **a** Patient with high baseline P1/P2 ratio. **b** Patient with low baseline P1/P2 ratio

Figure 3 shows an illustrative example of the time courses of P1 and P2 amplitude during infusion test for a single patient. The rise in the mean ICP during infusion resulted in an increase in amplitude of both P1 and P2 (*p* < 10^−6^), with a visibly larger change for P2: up to 6.22 [4.44–8.35] mm Hg mm Hg from baseline amplitude of 3.52 [2.44–4.49] mm Hg vs. 4.23 [2.7–4.74] mm Hg from baseline of 2.51 [1.44–3.56] mm Hg for P1. While baseline P1/P2 ratio varied between patients, with some patients exhibiting pronounced P1 (P1/P2 > 1) and some already showing increased P2 (P1/P2 < 1), the P1/P2 ratio during plateau fell to around 1 and below. Time courses of both amplitudes were strongly correlated with changes in the mean ICP; however, slightly higher correlation was observed for P2 (group-averaged correlation coefficient equal 0.98 [0.93–0.99] vs. 0.95 [0.82–0.96] for P1). The magnitude of decrease in P1/P2 ratio between baseline and plateau phases was not correlated with either baseline ICP, change in ICP, or elasticity estimated based on the CSF dynamics model. It showed weak although statistically significant, inverse correlation with baseline P1/P2 ratio (*R* = –0.38, *p* = 0.03; Fig. 4), with largest changes observed in cases where baseline ICP waveform contained P1 dominating over P2. Figure 5 shows an example of changes in ICP waveform between baseline and plateau for patients with high and low baseline P1/P2 ratio.

### Comparison of three estimates of cerebrospinal compliance

**Fig. 6:**
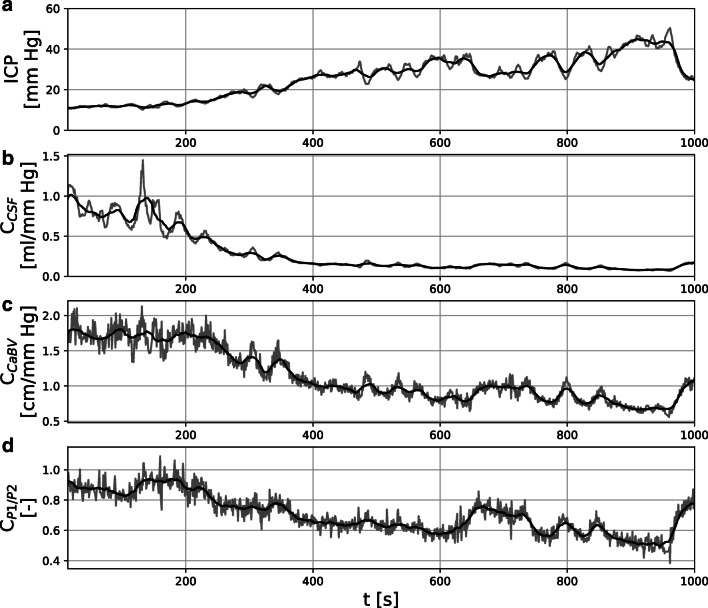
Illustrative example of time courses of compliance estimates for a single patient. Full pulse-by-pulse time courses are presented as grey lines, while 30-pulse moving averages are presented as black lines. **a** Mean intracranial pressure (ICP). **b**–**d** Compliance estimates obtained with **b** CSF dynamics model (C_CSF_), **c** cerebral blood volume model (C_CaBV_) and **d** P1/P2 peak ratio of ICP pulse waveform (C_P1/P2_)

Figure 6 shows an illustrative example of the time courses of compliance estimates obtained with each of the three methods for a single patient. Compliance estimates were positively and statistically significantly (*p* < 0.05) correlated: 0.94 [0.88–0.97] for C_CSF_ vs. C_CaBV_, 0.77 [0.63–0.91] for C_CSF_ vs. C_P1/P2_, and 0.68 [0.48–0.91] for C_CaBV_ vs. C_P1/P2_. Similarly, compliance estimates C_CaBV_ and C_P1/P2_ showed inverse correlation with the mean ICP, although the correlation was stronger for C_CaBV_ (−0.82 [−0.71–−0.86]) than C_P1/P2_ (−0.71 [−0.46–−0.79]); as C_CSF_ was calculated using the mean ICP itself, this pair of parameters was not compared.

Between baseline and plateau phase of the infusion test, group-averaged mean ICP increased from 13.4 [9.5–16.5] to 22.6 [18.7–28.0] mm Hg. Accordingly, all three methods showed a statistically significant decrease in compliance estimates between baseline and plateau (Wilcoxon signed rank test *p*-value < 10^−4^) but with a varying degree of absolute changes, with the largest change observed for the values obtained with the CaBV model (baseline vs. plateau, 1.16 [0.71–1.75] cm/mm Hg vs. 0.72 [0.50–1.00] cm/mm Hg), smaller for the CSF dynamics model (0.67 [0.37–1.16] ml/mm Hg vs. 0.27 [0.17–0.51] ml/mm Hg)), and smallest for the P1/P2 ratio (0.69 [0.58–0.90] vs. 0.57 [0.46–0.74]). Decrease in compliance estimates relative to baseline was 46.3% [36.5–65.1%], 41.0% [30.8–48.4%], and 16.4% [7.3–27.9%] for the C_CSF_, C_CaBV_, and C_P1/P2_ method, respectively.

## Discussion

The analysis of ICP pulse waveform, which relies on the assumption that variations in the pulsatile component of ICP reflect the pressure response to volume changes induced by the flow of cerebral blood in each cardiac cycle [2], has been the basis of various indirect methods of compensatory reverse estimation proposed over the years, such as the amplitude–pressure curve [31], high-frequency centroid [28], or RAP index [11]. The P1/P2 ratio, although suggested as a measure of cerebral compliance in the 1980s, so far has not been explored in much detail. A study by Cardoso et al. [6] showed that hyperventilation-induced reduction in the mean ICP is accompanied by a decrease in P2 and relatively small change in P1, while similar reduction caused by head elevation or CSF withdrawal has little to no effect on the shape of the pulse waveform. It was suggested that this would indicate the dependence of P2 on cerebral compliance. Results of the present study are in accordance with previously reported observations [6, 22]. Increase in the mean ICP caused by infusion of volume into the CSF space produced a noticeably larger change in the amplitude of P2 for similar baseline amplitudes of P1 and P2, resulting in decreases in the P1/P2 ratio. Individual time courses of the P1/P2 ratio followed the time courses of compliance estimated based on the CSF dynamics model relatively well, with mean correlation coefficient at the level of 0.75.

The differences in the time courses can be explained by the dependence of C_CSF_ on the trend in the mean ICP, used both in the estimation of elasticity and P_0_ and further calculation of compliance, as the P1/P2 ratio is calculated only from the shape of the ICP waveform and on a pulse-by-pulse basis. However, this effect was partially mitigated by the use of moving averages in correlation analysis. The slightly weaker correlation with values of C_CaBV_ may in turn be the effect of inclusion of another signal, CBFV, in the model. Although the extent of decrease in compliance varied between methods, largely due to varying range of values obtained with each approach, all three methods allowed for statistically significant differentiation between the ‘high’ and ‘low’ compliance state associated with baseline and plateau phases of the infusion test. However, as the CaBV and P1/P2 methods examine the ICP pulse waveform resulting from unknown volume load, they are indirect measures that cannot be translated to units of compliance, and straightforward comparison of the values is not feasible. Table 1 outlines major features and limitations of each method of compliance estimation considered in this study.

**Table 1:** Comparison of three methods of cerebrospinal compliance estimation

Characteristic	Method		
	Based on the model of CSF dynamics	Based on the evaluation of CaBV from TCD recordings	Based on the analysis of the P1/P2 ratio of ICP pulse waveform
Units	Absolute [ml/mm Hg]	Express compliance per unit of cross-sectional area of insonated vessel [cm/mm Hg]	Relative changes only [dimensionless]
Assessment	One-off measurement (based on a recording from entire infusion test)	Continuous but limited by positioning of TCD probes	Continuous
Accuracy	Good	Relative changes only	Relative changes only
Availability	Always when access to CSF space is possible	Requires TCD monitoring	Requires that P1 and P2 are detectable in the ICP pulse waveform
Additional requirements	Requires invasive ICP measurement	Requires invasive ICP measurement (with good quality of pulse waveform)	Requires invasive ICP measurement (with good quality of pulse waveform)

One advantage of assessing cerebral compliance based on the P1/P2 ratio is the fact that it does not depend on external volumetric manipulation necessary to derive the pressure–volume curve. In principle, the method also does not require any additional signals beside ICP that are needed to estimate changes in CBV. It should be noted, however, that the major difficulty lies in reliable, fully automated detection of peaks P1 and P2. A number of algorithms of often impressive complexity have been proposed over the years for the task of ICP peak identification [5, 14, 17, 30]. Those methods remain a relatively recent development in the continuously advancing field of biomedical signal processing, and despite promising results reported so far, they are yet to find acceptance in the medical community and most importantly validation in large scale trials that would allow for introduction to standard clinical practice. Still, the ICP pulse waveform exhibits large inter- and intrapatient differences, with varying height and prominence of characteristic peaks that may not all be visible even in patients with normal compliance. Whereas the detection of P2 is usually possible despite pathological rounding of the pulse, the detection of P1 becomes unachievable. Incorporation of the ABP signal may improve general peak identification accuracy by allowing for better differentiation between P1 and P2 candidates. Nevertheless, the P1/P2 ratio approach relies heavily on the performance of the underlying detection algorithm, and that in turn requires that the signal is recorded with sufficiently good quality of ICP pulse waveform.

In the present study, in order to provide as reliable as possible assessment of the feasibility of using the P1/P2 ratio as an estimate of cerebrospinal compliance, P1 and P2 candidates identified by the algorithm using both the analysis of ICP pulse waveform and its relationship with ABP (where available), CBFV, and CaBV signals were manually reviewed and corrected in cases of insufficient detection accuracy. In order to consider validating this approach in the clinical setting, further work is required to refine the prototype and develop it into a fully automated method of peak identification. Moreover, a comprehensive solution would not only need to be capable of analysing in real time the large variety of ICP pulse waveform shapes encountered in patients with intracranial pathologies but also detecting artefacts and selecting pulses where the peaks, particularly P1, become impossible to distinguish due to the rounding of the waveform and not due to the low quality of the signal.

On the other hand, assuming that the peaks are identified reliably, it is theoretically possible to compare the results between separate measurements and between individuals. A previous study by Fan et al. [15] attempted to use the P2/P1 ratio (as opposed to the P1/P2 ratio used in the present study) as a predictor of disproportionate increases in ICP in TBI patients. Elevated P2 amplitude was signified by P2/P1 ratio equal to or exceeding 0.8. The study showed that the P2/P1 ratio, while not a unique predictor of intracranial hypertension episodes, was significantly higher in the group exhibiting ICP increases. A different study in paediatric hydrocephalus patients confirmed the applicability of the P2/P1 ratio in the identification of intracranial hypertension and assessment of the response to shunting [3]. It should be noted that other works focused on the classification of ICP pulse waveform patterns [14, 26] suggested that decrease in compliance is associated with P2 visibly dominating over P1. A threshold ratio of 0.8 could therefore potentially lead to the inclusion of normal waveforms in the elevated P2 group and influence the results.

Ultimately, the use of the P1/P2 ratio as an indicator of decreased buffering capacity of the cerebrospinal system would require further investigation with regard to the correlation between the value of P1/P2 ratio and the clinical status of the patient, as this measure cannot be directly converted to units of compliance. Given earlier reports on the relationship between measures of compliance and compensatory reserve and outcome in TBI patients [25, 27, 28, 32], combined with the possibility of continuously recording the ICP waveform in modern neuro-critical care units, this avenue of study seems nonetheless to be a promising one.

### Limitations

Compliance estimation based on the evaluation of changes in CBV as used in this study is based on a number of assumptions, including constant diameter of insonated vessels during TCD recording. Due to unknown cross-sectional area of the vessels, C_CaBV_ values cannot be calibrated in units of compliance. The same applies to the P1/P2 ratio as changes in the ICP pulse waveform are analysed as a response to unknown blood stroke volume. Consequently, direct comparison between those two methods and the ‘gold standard’ approach based on the model of CSF dynamics was limited to the correlation between the time courses.

Moreover, this study was performed as a retrospective analysis of infusion test recordings of the ICP signal with the additional requirement of availability of simultaneously collected CBFV. As TCD measurements are not part of routine clinical investigation in NPH patients, the number of available recordings was limited, and given the size of the study group, the results of this study should be regarded as preliminary. Considered recordings were not collected with the explicit purpose of analysing the ICP pulse waveform in detail, which led to a relatively high percentage of cases (almost 30% of the initial dataset) excluded on the basis of low signal quality.

However, even assuming sufficiently high quality of the signal, the visibility of peaks remains a significant limitation. On the other hand, pulses with indistinguishable P1 due to the rounding of the waveform constitute a separate class of signals. Whereas they do not allow for monitoring of relative changes over time, they could be incorporated as a form of low compliance indicator. This could be a viable solution especially in long-term monitoring aimed at detecting decreases in compensatory reserve, where peak visibility is expected to vary.

## Conclusions

Apart from the ‘gold standard’ method, compliance of the cerebrospinal fluid system may be evaluated using pulse waveform of ICP and TCD recordings of blood flow velocity in the cerebral arteries. The latter two methods agree with the ‘gold standard’ approach based on volume addition. It potentially opens new perspectives for continuous brain compliance monitoring in various clinical scenarios.

## List of abbreviations

ABP: Arterial blood pressure [mm Hg]
CBFV: Cerebral blood flow velocity [cm/s]
CBV: Cerebral blood volume [ml]
CSF: Cerebrospinal fluid
ICP: Intracranial pressure [mm Hg]
NPH: Normal-pressure hydrocephalus
TBI: Traumatic brain injury
TCD: Transcranial Doppler
